# The Pediatric Trochlear Migraine: Diagnostic and Therapeutic Implications

**DOI:** 10.3390/jcm11102826

**Published:** 2022-05-17

**Authors:** Vincenzo Raieli, Federica Reina, Daniela D’Agnano, Giovanna Martina Nocera, Mariarita Capizzi, Francesca Marchese, Vittorio Sciruicchio

**Affiliations:** 1 Child Neuropsychiatry Unit ISMEP, ARNAS Civico, 90134 Palermo, Italy; marchesefrancescaa@gmail.com; 2 Child Neuropsychiatry School, University of Palermo, 90128 Palermo, Italy; federey01@gmail.com (F.R.); giovannamartinanocera@gmail.com (G.M.N.); mariaritacapizzi93@gmail.com (M.C.); 3 Children Epilepsy and EEG Center, PO, San Paolo ASL, 70132 Bari, Italy; daniela.dagnano@gmail.com (D.D.); vittorio.sciruicchio@asl.bari.it (V.S.)

**Keywords:** trochlear region, migraine, primary trochlear headache, pediatric headaches, international headache classification, children

## Abstract

Trochlear Migraine has been recently described as the concurrence of strictly unilateral migraine and ipsilateral trochleodynia with relief of migraine after successful treatment of trochleodynia. This disorder has been interpreted as “cluster-tic syndrome” or “seizure-triggered migraine”. Trochlear Migraine is unrecognized and rarely described in childhood. The aim of this study is to review the few cases of Trochlear Migraine reported in the literature in addition to the cases observed in our clinical experience. In particular, our cases showed recurrent attacks of severe and pulsating headache associated with nausea, vomiting, phonophobia, photophobia, and strict trochlear localization of pain. They often presented with alternating side attacks. Therefore, we suggest that the term “Trochlear Migraine” should be reserved for clinical migraine attacks strictly localized in the trochlear region, and we assume that the excessive increase in descriptions of new primary headache syndromes, according to the International Classification of Headache Disorders, can be probably be ascribed to the common physiopathological mechanisms characterizing these forms of migraine.

## 1. Introduction

Trochlear pain or trochleodynia describes different disorders characterized by pain in the superomedial side of the orbit. This site involves the supra and infra trochlear nerves. The pain develops in the trochlear area through nociceptive afferents, activates the trigeminal nerve, and causes different types of headaches [1].

Some authors [2,3,4] have previously introduced two new primary headache types: “Primary Trochlear Headache (PTH) and Trochlear Migraine (TM)”.

According to the 3rd Edition of the International Classification of Headache Disorders (ICHD-3), Primary Trochlear Headache (PTH; 11.3.4) describes a unilateral pain developing from trochlear and temporo-parietal regions, showing pressure-like quality, moderate intensity, almost continuous, exacerbating by supraduction of the symptomatic eye [5]. It is treated with local steroid inoculation, in the absence of inflammatory signs and orbital or systemic disease. Primary trochlear headache often coexists with other primary headaches, mainly migraine [6].

Yanguela et al. [2] first coined the term “Trochlear Migraine” (TM) referring to the concurrence of this unilateral trochlear pain and ipsilateral migraine attacks, for which the appearance of the first pain worsened the second one, and the unilateral trochlear pain relief improved the migraine attacks. This disorder has been interpreted as similar to “cluster-tic syndrome” or “seizure-triggered migraine” [2]. Therefore, the two nosographic pictures would concur in the same patient, one influencing the other disorder during the clinical course.

Initially, “Trochlear Migraine” has been described only in the adult age and no pediatric cases have been previously reported in the literature [7,8]. However, we have recently described a migraine child with trochlear localized pain, questioning the use of the term “Trochlear Migraine” to indicate the two co-occurring pathologies. According to our interpretation, this was due to a form of migraine with atypical localization and typical characteristics of migraine, the “Pure Trochlear Migraine”, which has been differentiated from Primary Trochlear Headache and Secondary Trochleitis [9]. Furthermore, to our opinion, other pediatric cases of that resemble possible “Pure trochlear migraine”, following the clinical description of PTH [10], have been also reported or included in mixed clinical series of adolescents and adults [11].

Therefore, we propose a short case series of Trochlear Migraine and a review of literature of cases with this atypical location of migraine in order to discuss diagnostic and therapeutic implications and to add further knowledge to Pure Trochlear Migraine (PTM) features in childhood.

## 2. Case Series

### 2.1. Case 1

**Figure 1:**
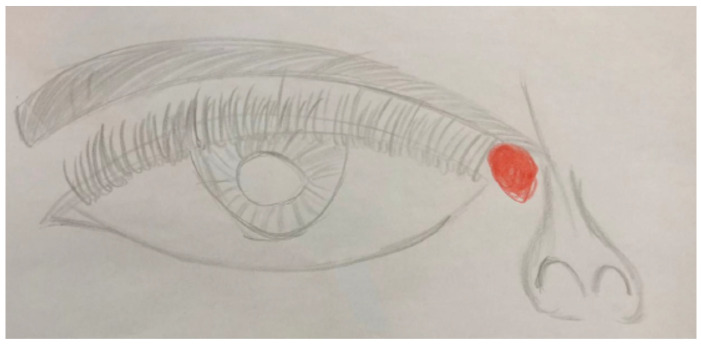
Pain localization pictured by child *n*.1 of 12-year-old.

A 12-year-old male was admitted to our observation for painful attacks at the level of the inner corner of the right eye with bulbar pain and irradiation of the pain in the periorbital site, other times with involvement in the right temporal site, sometimes left and sometimes bi-temporal with irradiation in the occipital area and often characterized by burning or throbbing pain, of severe intensity with VAS of 9 (Visual Analogisc Scale) with gradual onset (often rising in the morning with a headache). The attacks were unresponsive to common analgesics, and benefited from compression. They were associated with phono and photophobia…(2–3). These episodes began at the age of 6 when they were less frequent and less intense and were responsive to paracetamol. For about two years, the frequency of episodes and the intensity have progressively increased. We performed Brain MRI that resulted normal (left turbinate hypertrophy). He weared corrective lenses for myopia and astigmatism.In his clinical history no other comorbidities were reported. There was a positive family history for menstrual migraine (mother) and migraine without aura. We prescribed a treatment cycle with flunarizine 5 mg/die. He had only three attacks in the month of February. The spontaneous drawing (Figure 1) accurately supports the child’s anamnestic narrative about the site of migraine pain.

We report the child’s drawing showing his infra-trochlear pain localization in Figure 1.

### 2.2. Case 2

An 11-year-old male, admitted to our pediatric headache center (Child Neuropsychiatry Unit -ISMEP Palermo) in March 2018, complained of several migraine attacks for two years with increasing frequency in the last six months. He showed episodic headache attacks (frequency of 3–4 attacks per month) with pulsating quality, severe intensity (VAS = 7–8), duration of several hours and alternating side, that were associated with nausea, photophobia, phonophobia, and mild unilateral cranial autonomic symptoms. Sleep gave good relief. The main atypical characteristic observed was that for several reported attacks, the pain location was strictly limited to the unilateral superior—inner angle of the orbit associated with tenderness to pressure over the infratrochlear area, without a radiating or swollen trochlea. The patient had a positive familiarity for migraine (mother). The neurological examination and instrumental exams (i.e., orbital TC) were negative. Considering the low frequency of attacks, we only prescribed symptomatic treatment for painful attacks with ibuprofen (10 mg/kg) and preventive therapy with Magnesium 300 mg/die with benefits for three months.

### 2.3. Case 3

The second case, admitted to the Child Neuropsychiatry Unit -ISMEP Palermo, was a 16-year-old female affected by recurrent unilateral alternating headache attacks in the last seven years. The quality of attacks was pulsating, with frequent trochlear localization, severe intensity (VAS = 8–9) associated with nausea, vomiting, photophobia, and phonophobia. The trochlea did not appeared swollen, she complained mid pain to pressure, mild lacrimation was present. The frequency of attacks was 2–3 episodes per month with a duration of several hours. The atypical location of pain induced severe fear in the parents and in the family physician. All the exams were negative. Familiarity was positive for migraine and epilepsy (mother). She responded to the Sumatriptan nasal spray 20 mg. No preventive treatment was prescribed for the low frequency of the painful attacks.

### 2.4. Case 4

**Figure 2:**
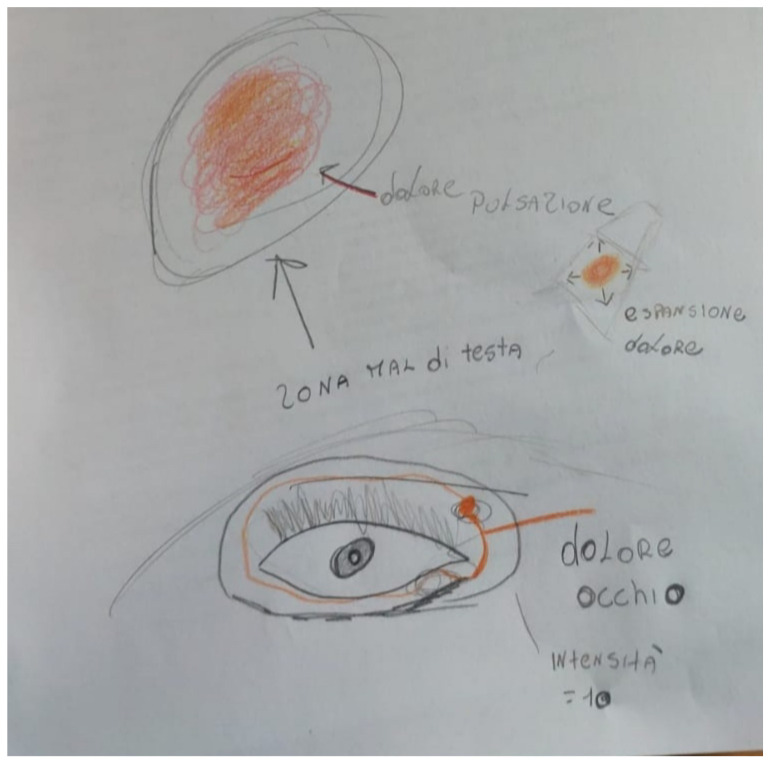
Pain localization pictured by child *n*. 4 of 11-year-old. dolore pulsazione—throbbing pain; zona mal di testa—headache area; espansione dolore—pain expansion; dolore occhio—eye pain; intensita—intensity.

An 11-year-old male, admitted to our pediatric headache center (Child Neuropsychiatry Unit -ISMEP Palermo) in February 2021, complained of several migraine attacks since May 2020 with increasing frequency in the last two months. He showed episodic headache attacks with a frequency of 1–2 attacks per week, pulsating quality, severe intensity (VAS = 8), duration from several hours to two days, strictly unilateral right-side, associated with nausea. No vomiting, photophobia, and phonophobia reported. The attacks had a pain location strictly limited to the unilateral superior–inner angle of the orbit, reduced by massage pressure over infra-trochlear area, with potential pain radiating to the temporal-occipital region. No increase of pain detected with ocular movements and responsiveness to ibuprofen. Positive familiarity for migraine (mother). Neurological examination and instrumental exams (Brain MRI) were negative. We prescribed Sumatriptan nasal spray 10 mg for the acute attack, and Magnesium and tryptophan amino acid with good relief. The spontaneous drawing (Figure 2) accurately supports the child’s anamnestic account of the site of migraine pain and, in addition, the child illustrates other pain characteristics such as intensity, quality of pain, and pain irradiation, making the drawing and account even more explanatory.

## 3. Clinical Features of PTM

After our first publication [9], other descriptions of PTM or probable PTM [10,11] have been added to the literature supporting the non-rarity of this clinical presentation in children. However, it should be specified that Sanchez Ruiz et al., defined their case as Primary Headache Trochlea [10] although the child described was an adolescent patient with a history of migraine, who showed episodic painful unilateral infratrochlear attacks associated with moderate photophobia as well as abdominalgia and incoercible vomiting, satisfying the criteria for migraine despite the atypical localization. Unfortunately, the authors did not describe other aspects regarding this potential case of “pure” TM and did not report the effects of local therapy on previous migraine attacks.

From the description of these cases [9,10,11] together with our case series, it is possible to define the main characteristics of this potential type of migraine: the initial infra-trochlear localization of pain in many attacks; the episodic frequency of the attacks; the duration of the attack must be >2 h; the pain is throbbing as in a typical migraine; frequent alternation of sides; the presence of associated autonomic signs such as nausea, vomiting (typical of a migraine attack); the reduction of pain at infra-trochlear compression, similar to the reduction of pain at temporal compression typical in children with migraine; the possible presence of local autonomic signs; irradiation of the episodes in the temporal-frontal area unilaterally, and possible painfulness in movements; the presence of typical migraine attacks in addition to those of PTM.: “In addition, the good response to 5 HT1 agonists (see Sumatriptan), specific drugs in the therapy of migraine further supports the diagnosis of migraine”.

These characteristics satisfy the diagnostic criteria for migraine, however, the PTM includes some typical characteristics of the involved pain site, such as tenderness to palpation over the infra-trochlear area (allodynia), the possible reduction of pain due to infrat-rochlear compression, and local autonomic signs closely localized in the orbit. Full well-being between attacks, benefit from the local steroid and anaesthetic treatment, and familiarity for migraine have also been reported. The tests performed for secondary forms of migraine are negative. Asking the child to draw the site of pain can be helpful for precise localization (Figure 1 and Figure 2).

In Table 1, we summarize the main clinical features of our four patients [10] and the Sanchez-ruiz et al.’s case report compared to the Yanguela et al. [2] cases defined as Trochlear Migraine.

## 4. Physiopathological and Anatomical Considerations

The peculiar aspect of PTM is the correlation of a specific localization with a clinical phenotype of migraine that involves more general functional systems, such as cyclic attacks, systemic autonomic disorders, reduced sensory tolerance, etc.

Migraine, despite being a pathology with main central physiopathogenetic mechanisms where among the main pathogenetic mechanisms is serotonergic modulation that from the nucleus raphe magnus (NRM sending specifically serotonergic projections to the cervical trigeminal complex (rich in 5 HT1 receptors) and modulates the ascending nociceptive trigeminal thalamic fibers [1,12], is closely related to peripheral information coming from the sensitivity of the head that can trigger the migraine attack, even from structures not innervated by the trigeminal nerve, due to the trigeminal-cervical overlap [1,12]. Therefore, it is not surprising that even atypical sites, such as the trochlear or nasal regions, if potentially irritated can trigger a migraine attack with pain mainly localized to this site but with generalized manifestations. The trochlea is a cartilaginous structure located in the superior-medial corner of the orbit, crossed by the tendon of the superior oblique muscles, and innervated by the sensory fibers of the ophthalmic nerve. Therefore, its sensory innervation can send nociceptive afferents to the trigeminal nerve and can thus constitute an activation point for migraine, with pain originating from the periorbital area that can radiate to the ipsilateral frontal site [13,14]. The reported cases, in fact, presented exemplary examples, where attacks with a more typical site (fronto-temporal) alternated with attacks at infra-trochlear site, due to the probable presence of trigger points in this site [9,10,12]. It is possible, in those cases with attacks strictly localized in the unilateral superior-inner angle of the orbit and no other main localizations, that there would be a peripheral locus of the genesis of migraine pain [13,14] which is especially alternating and exclude a secondary peripheral cause.

This topographic localization of pain in migraine patients is probably less described because the trochlear examination is not systematically performed in patients with migraine [2]. A previous study suggested that a large group of unilateral migraine patients (80%) perceived a referred pain from stimulation of the trochlear region by the involvement of local myofascial structures during free headache interval [14]. The authors speculated that nociceptive stimuli of the trochlea could trigger and perpetuate other types of primary headaches.

In fact, migraine pain mostly develops within the innervation territory of the first branch of the trigeminal nerve [12]. Moreover, the topographical description of migraine is often undervalued. These considerations are supported by a recent interesting report describing 43 patients with trochlear pain of which two subjects had side-alternating pain, and seven and six cases had respectively nausea and photophobia [15].

It has been hypothesized that co-occurrence of migraine and trochleodynia, both sending painful inputs via the first branch of the trigeminal nerve to the caudalis trigeminal nuclei overload it with spatial and temporal painful signal summation provoking an increase of migraine [10]. This hypothesis has been advocated to explain the efficacy of local treatment in Trochlear Migraine, as defined by Yanguela et al. [2], but has not clarified the trochlear migraine attacks with alternating sides.

Non steroid anti-inflammatories treatment normally causes a significant improvement of symptoms through decreasing nociceptive effects on the trigeminal nerve.

Furthermore, the Spanish researchers’ criterion [3,4] of pain reduction by local steroid injection was not always met in another clinical report [8] (no effect in 26.7%), and the same group recently affirmed the non-specific response of this treatment in the primary headaches [4]. Therefore, it is probably that many adults with Primary Trochlear headache complain of migraine attacks [11].

## 5. Differential Diagnosis

Many clinical syndromes are associated with localized pain in the orbital region [16,17,18,19,20,21,22]. Previously, some authors suggested that orbital pain may also be categorized based on differences seen in pain characteristics, degree of severity, and type of onset [21,22]. All sensation coming from the orbit is transferred along with the V1 distribution, while the ocular-motor nerves have been presumed to be strictly motor in function with no pain receptors or pain pathways [19,20]. However, little is known how pain starting from orbital receptors reaches the ophthalmic division of the trigeminal nerve (V1) and travels to the descending spinal trigeminal nucleus.

The PTM must be differentiated by primary trocleodynia or primary trochlear headache (PTH), trigeminal autonomic headaches (TACs) with orbital localization, and secondary disorders provoking pain in the trochlear region [22].

The PTH may be diagnosed in presence of strict unilateral fixed or sometimes bilateral localization, the temporal pattern is almost always continuous and not episodic, scarce vegetative symptoms are associated with headache, the trochlear region can be edematous, and the presence of painful ocular movements may not be possible to investigate (however only 50% had this symptom in a recent case-collection) [15]. However, PTH shares some features with PTM: the side of the pain and the soreness to local pressure in infra-throclear region. A familial history of migraine may help to differentiate the two disorders.

Furthermore, we want to underline that recent data [11] of a clinical sample of PTH (divided into episodic and continuous PTH in relation to the temporal pattern of pain, including adolescents) showed that many episodic PTH attacks triggered migraine or tension attacks. In the majority of the cases, the headache started from the head and was responsive to the typical preventive therapy for primary headaches [11]. So, the question may arise whether these PTHs were nothing more than migraine sufferers with atypical location or irradiation. On the other hand, there are several data that support the peripheral contribution of triggers in neighboring regions causing the migraine attack [23] and whose treatment, see botulinum toxin, is effective [24].

Other primary headaches (TACs) [25] or nummular headaches [26], and neuralgias (infratrochlear and supraorbital neuralgias) [27,28] are similar in size of pain, local site, or presence of cranial local autonomic and neurovegetative symptoms, and could be excluded based on the temporal pattern (daily attacks versus pluri-daily attacks, episodic versus continuous or intermittent-remittent), alternating shift versus fixed side, quality of pain, behavioral features etc. Secondary causes (trochleitis) [22], including several disorders such as systemic inflammatory diseases, Brown syndrome [29], orbital myositis [30], thyroid ophthalmopathy [31], Tolosa-Hunt syndrome [32], may be ruled out by the temporal pattern, alternating side, the positive physical and/or neurological examination, blood tests, ophthalmologic consult, and neuroradiological imaging [11,22].

Referring to the role of autoimmunity in triggering the disorder, Oijha et al. [11], showed that, in their patients, the detection of autoantibodies, that is antinuclear antibodies and antithyroid antibodies, was strictly associated with refractoriness to migraine prophylaxis and an improvement on treatment with immunosuppressant.

In Table 2 we report the main features of PTH, PTM and Supra/infratrochear neuralgias.

## 6. Nosographic Considerations

According to the Spanish researchers [2], the first coined term “Trochlear Migraine” would be used to indicate the association between two distinct disorders, PTH and Migraine, occurring together, where the treatment of the first ameliorates the second disorder [1]. Instead, we proposed that the definition of “Pure Trochlear Migraine” should be reserved for a clinical migraine attack strictly localized in the trochlear region, similar to the “Nasal Migraine” term, used by the same group to describe migraine attacks strictly localized in the nasal region [33,34]. On the other hand, other authors described that the treatment of PTH was effective on another type of primary headache, chronic paroxysmal migraine [35]. However, in this case the authors should have coined the term “Trochlear Paroxysmal Hemicrania”, in the light of the interpretation suggested for “Trochlear Migraine” by Yanguela et al. [2]. Furthermore, as reported by the same group of researchers, the use of local infratrochlear treatment with steroids and anaesthetics is also effective in primary headaches where there is no diagnosis of PTH [36], so this treatment is non-specific and not always effective in PTH [4]. However, the detection of migraine pain in the trochlear area may suggest a local treatment in that site, in the presence of failure to respond to usual pharmacological treatments, similarly to the local use of steroid courses in the occipital area, in chronic migraine [37]. Also, it is possible to hypothesize that a peripheral treatment of this site could be useful in migraine prophylaxis therapy.

Regarding the clinical features of this rare headache, these resemble the characteristics of the Primary Trochlear Headaches with the following salient differences:the pain was often side-alternating but not side-locked, and potentially radiating in other cephalalgic localization;

The temporal pattern of the episodic attacks was recurrent and not continuous; our patients presented important vegetative symptoms associated with headache, very rarely described in PTH [2,3];

### Migraine Familiarity; Pain Relief with Rest and Sleep

However, the PTM, to our opinion, mainly suggests some clinical considerations on the risk related to the ICHD-3 classification. Therefore, a final relevant question could be whether the ICHD-3 classification of primary headaches [4] could be responsible for the excessive and increasing descriptions of new primary headache syndromes based on particular diagnostic criteria. For instance, new syndromes are defined in relation to atypical topography (see PTH [2] or idiopathic rhinalgia [33,34]), the direction of pain (see hemicrania fugax [38]), the size of painful area (see nummular headache [26]), or with associated autonomic symptoms (see red ear syndrome [39]).

Alternatively, a more restrictive approach could be adopted to better classify the headaches in relation to the well-defined pathophysiological model of cranial pain, based on our actual scientific knowledge where the single case confirms or disconfirms the suggested model. If we consider this second hypothesis, it could be less useful to identify a disorder in relation to the localization or other clinical features.

## 7. Conclusions

The “Pure Trochlear Migraine“ is a rare condition with mixed association of local topographic peculiar signs and typical criteria for migraine attacks. In our opinion it is important that physicians take in account the possibility that migraine may occur in atypical sites such as the trochlear site in the absence of inflammatory or other pathologies as evidenced by the recent International Classification of Orofacial Pain (ICOP) [40] which identifies in group 5 primary headache with exclusive localization in the lower part of the skull. Keeping in mind this diagnostic hypothesis could avoid excessive and invasive diagnostic work-up.

We suggest to use the term “Trochlear Migraine“ in unambiguous manner with respect to the coined Yanguela’s definition [2]. Nowadays, few reports, mostly of pediatric cases, are available and more evidence is needed in order to clarify the boundaries of “Pure Trochlear Migraine” so that this could be considered a distinct type of primary headache.

From a nosographic point of view, we discuss that we should pay attention to the spreading and increase in descriptions of new primary headaches, which could impact on the ICHD-3 classification of primary headaches [5], based on syndromic criteria, and in terms of diagnostic effectiveness.

A more effective approach could instead be to focus on the defined pathophysiological model of cranial pain to which clinical features could be attributed.

## Figures and Tables

**Table 1:** Pediatric trochlear migraine–our four cases report, pediatric case from literature and Yanguela’s Trochlear Migraine cases.

Clinical Features	1 (Raieli Unpublished Case	2 (Raieli Unpublished Case	3 (Raieli Unpublished Case	4 (Raieli Unpublished Case	1 (Yanguela et al., Neurology 2002)	2 (Yanguela et al., Neurology 2002)	3 (Yanguela et al., Neurology 2002)	Sanchez-ruiz et al. 2020
Sex	M	M	F	M	F	F	F	F
Age at diagnosis trochlear pain	12	11	16	11	?	?	?	13
Age at onset of migraine, y	6	10	11	10	8	15	17	?
Migraine subtype	Ep MwA	Ep MwA	Ep MwA	Ep MwA	Ch	Ep	Ch	MwA
Location of migraine pain	Troch.	Troch/fronto-temp	Troch/front	Troch	R H	L H	L > R H	?
Age at onset trochlear pain	6	Not specified	1 years	10	39	49	56	13
Side trochlear pain	Alternanting Dx > sx	Alternanting	Alternantindx	Unilateral dx	U	U	U	U sx
Quality troclear pain	Puls./pre	Puls.	Puls.	Puls.	Puls.	Squez.	Sand	?
Temporal pattern of active pain period	Recurrent episodes	Recurrent episodes	Recurrent Episodes	Recurrent episodes	Cont.	Cont.	Cont.	Cont
Intensity of trochlear pain (not included excerbations)	7–9	7–8	7–8	8	4	4–5	3–4	7
Photo/phonophobia associated to trochlear pain	+	+	+	−	−	−	−	+
Nausea associated to trochlear pain	+	+	+	+	−	−	−	+
Vomiting associated to trochlear pain	+	−	+	−	−	−	−	+
Diplopia	−	−	−	−	+	−	−	−
Trigger trochlear for migraine attacks	Not applicable	Not applicable	Not applicable	Not applicable	+	−	−	−
Response to local steroid injection	Not applicable	Not applicable	Not applicable	Not applicable	+	+	+	+
Response to triptans	No report	No report	+	+	No report	No report	No report	No report
Preventive treatment	+	+	No	+	No report	No report	No report	No report
Familiarity for Migraine	+	+	+	+	No report	No report	No report	No report

?, not specified; Ch, chronic migraine; Ep, episodic migraine; RH, right hemicranias; Lh, left hemicranias; Troch., trochlear region; U, unilateral; B, bilateral; A, alternating; Puls, pulsating pain; Dull, dull ache, Squez, squeezing pain; Sand, sand pain; Pre, pressure-like pain; Cont, continuous; R, recurrent attacks; NA, not applicable.

**Table 2:** Clinical profile of primary trochlear headache (PTH)–pure trochlear migraine (PTM)—supra/infraorbital neuralgias.

	Primary Trochlear Headache	Pure Trochlear Migraine	Supra/Infratrochlear Neuralgias
Age at onset	Adult>	pediatric	variable
Pain location	trochlear	trochlear	Supra/medial infratrochlear
Side location	unilateral	Unilateral- alternating side, bilateral	unilateral
Other cephalalgic side	sometimes Frontal-temporal	Frontal-temporal	frontal
Temporal pattern	Chronic	episodic	Continue > episodic
Quality of pain	variable	pulsanting	Sharp paroxysmal /dull
Duration of painduring symptomatic period	daily	hours	daily
Intensity Pain	Moderate/severe	Moderate/severe	severe
Allodynia	−	+	+
Local symptoms	*−*	*+*	*−*
Vomit	*−*	*+*	*−*
Nausea	*+−*	*+*	*−*
Phonophobia	*−*	*+*	*−*
Photophobia	*+−*	*+*	*−*
Painful ocular movements	*+*	*−+*	*−*
Selective tenderness on the trochlear area	*+*	*−+*	*+*
Other triggers	*?*	*Foods menstrual cycle etc.*	*trauma*
Response to local injection of corticosteroids within 48 h	*+*	*?*	*Anestethic blockade*
Radiological findings	*no*	*no*	*possible*
Secondary causes	*no*	*no*	*Trauma/compression*
Triptans response	*−*	*+*	*−*
Preventive oral treatment	*−*	*+*	*+−*

+: positive response; −: negative response; +−: variable, ?: not reported.

## Data Availability

The data are available in the clinical files.

## Abbreviations

Primary Trochlear Headache: PTH; Trochlear Migraine: TM; International Headache Classification: HIS.
